# Zimbabwe’s national third-line antiretroviral therapy program: Cohort description and treatment outcomes

**DOI:** 10.1371/journal.pone.0228601

**Published:** 2020-03-02

**Authors:** Cleophas Chimbetete, Tinei Shamu, Olivia Keiser

**Affiliations:** 1 Newlands Clinic, Harare, Zimbabwe; 2 Institute of Global Health, University of Geneva, Geneva, Switzerland; University of KwaZulu-Natal, SOUTH AFRICA

## Abstract

**Background:**

In 2015, Zimbabwe introduced third-line antiretroviral therapy (ART) through four designated treatment centers; three government clinics in Harare and Bulawayo, and Newlands Clinic (NC), operated by a private voluntary organization in Harare. We describe characteristics of patients receiving third line ART and analyzed treatment outcomes in this national programme as of 31 December 2018.

**Methods:**

We described the population using proportions for categorical variables, and medians and interquartile ranges for continuous variables. Patients from NC, where data were more complete, were followed from the date of starting third-line ART until death, transfer, loss to follow up or 31 December 2018.

**Results:**

A total of 209 patients had ever received third-line ART: 124 at NC and 85 from the three government clinics. HIV genotype results were available for 89 (72%) patients at NC and fourteen (16.5%) patients in the government clinics. Median duration of third line ART (years) in the government clinics was 2.3 (IQR:0.6–3.4), 1.3 (IQR: 0.7–1.7) and 1 (0.6–1.9). Of the 67 patients who received third line ART in the government clinics for at least six months, 53 (79%) had most recent viral load (VL) < 1000 copies/ml. Data on other treatment outcomes from government clinics were incomplete.

From NC: a total of 109 (88%) patients were still in care, 13 (10.5%) had died and 2 (1.5%) were transferred. Median duration of third-line ART was 1.4 years (IQR: 0.6–2.8). Among the 111 NC patients who had received third-line ART for at least 6 months, 83 (75%) had a VL <50 copies/ml and 106 (95.5%) had a VL <1000 copies/ml.

**Conclusion:**

Our findings demonstrate that, with comprehensive care, patients failing second-line ART can achieve high rates of virological suppression on third-line regimens. There is need to decentralize the provision of third-line ART in Zimbabwe. More needs to be done to improve completeness of data in the government clinics.

## Background

Zimbabwe has one of the highest burdens of HIV infection in the world with an adult prevalence of 14.2% and approximately 1.4 million people living with the infection [2016 Zimbabwe HIV Estimates]. As the national programme continues to grow, it is expected that the numbers of patients failing current first-line non-nucleoside reverse transcriptase inhibitor (NNRTI)-based antiretroviral therapy (ART) and needing second-line protease (PI)-inhibitor-based ART will increase [1]. With more and more people receiving PI-based second-line ART, which is more difficult to adhere to due to the higher pill burden and more toxicity, we anticipate that the need for third-line ART will increase. While the availability of second-line ART is expanding in resource limited settings (RLS), only a few countries have widely available treatment options for patients who fail both NNRTI and PI-based combinations due to the high cost and complexity of implementation [2]. The World Health Organisation (WHO) has recommended that national programs must make third-line ART available [3]. Third-line ART regimens should include medicines such as etravirine (NNRTI), boosted darunavir (PI) and dolutegravir (Integrase inhibitor). Recommended approaches to third-line therapy require access to routine HIV viral load (VL) monitoring and genotypic resistance testing [3,4].

The provision of third-line ART in resource limited sub-Saharan Africa (SSA) has many challenges. Access to routine VL measurements among patients on ART is low and this leads to late diagnosis of ART treatment failure [5]. Previous studies have shown that the majority of patients failing second-line ART have not acquired protease inhibitor resistance mutations [6–8]. Poor adherence has been shown to be the major reason for second-line ART failure [9,10]. Unfortunately, access to HIV drug resistance (HIVDR) testing is poor and hence accurate diagnosis of second-line failure in SSA is difficult. Furthermore, before third-line commencement, patients should be adequately counselled on the need for optimum adherence and this requires substantial time which is not always available because of the high patient volumes and low numbers of healthcare workers.

Documentation of outcomes of third-line ART in RLS is scarce, yet very important to enable improvement of patient care. Due to the high costs of third-line regimens it is crucial to assess the effectiveness of these regimens to ensure optimal use of the limited resources. A few studies have reported encouraging early treatment outcomes for patients receiving third-line ART in RLS [8,11–13]. Results have shown that patients who switch to third-line ART have good early treatment outcomes and are able to suppress their VL despite the high level of ART resistance observed before third-line initiation [12]. Data on long term outcomes of over a year for patients receiving third-line ART are not yet available in sub-Saharan Africa.

We set out to describe the cohort of patients receiving third-line ART in Zimbabwe as well as assess treatment outcomes. We describe the following: demographic characteristics of patients, proportion of patients who were still in care, died and those lost to follow up. We further report on the virological outcomes among patients who had access to routine VL monitoring at one of the main treatment centers. We have previously reported week 24 third-line treatment outcomes for patients receiving care at Newlands Clinic (NC) [8].

## Methods

### Study setting

The study was conducted at the four designated national third-line ART treatment centers. Three of the centers are government run opportunistic infections (OI) clinics and the fourth center, NC, is operated and funded by a private foundation. Three of these centers, (Newlands Clinic, Harare Central Hospital and Parirenyatwa Central Hospital) are in the capital city, Harare. The fourth center is in the second largest city, Bulawayo. All four clinics use standard national HIV treatment guidelines for the care of patients.

Unlike the other three centers, NC has had an effective electronic monitoring system from its inception. Furthermore, the clinic provides HIV genotyping, six monthly routine viral load monitoring, and other necessary laboratory and radiology services at no cost to the patient. Details of the model of care are published elsewhere [14].

### Treatment regimens

Following the WHO recommendations, Zimbabwe adopted a public health approach to its HIV program using standardized treatment regimens. Since the inception of the HIV treatment program in 2004, there have been several guideline changes in when to start patients on ART and what regimens to use. Until beginning of April 2019, adult first line regimens included an NNRTI (Nevirapine or Efavirenz) plus two nucleoside / nucleotide reverse transcriptase inhibitors (N(t)RTIs). In April 2019, the preferred first-line regimen was changed to a dolutegravir-containing regimen as recommended by WHO. Second line regimens include a ritonavir boosted PI (Lopinavir or Atazanavir) and two N(t)RTIs. All patients received triple therapy for the management of HIV infection except earlier in the program (2004–2015) when single dose nevirapine or nevirapine plus zidovudine dual combination was used for prevention of mother to child transmission (PMTCT).

Third-line ART was introduced in Zimbabwe in 2015 through four designated national third-line treatment centers. All the four centers are designated specialist HIV treatment clinics with access to laboratory and radiology support. Patients failing second-line ART are transferred to the nearest third-line center by their treating physicians. These patients are supposed to receive at least three months of enhanced adherence support before being offered HIVDR testing. National standard operating procedures recommend that patients must have confirmed good adherence to second-line ART to become eligible for HIVDR testing. The Stanford HIV Drug Resistance Database is used to assess resistance to ART. HIVDR testing is not available in the public sector and hence patients need to access testing in the private sector at a prohibitive cost. Second-line treatment failure is confirmed by the presence of any level of resistance to atazanavir and lopinavir. Third-line ART for adults and children is composed of boosted darunavir, dolutegravir / raltegravir (raltegravir was used between 2015 and 2017 before dolutegravir availability) and sometimes two additional N(t)RTIs (based on the HIVDR test results).

Monitoring for ART treatment success was previously predominantly done using clinical events and routine CD4 measurements. Routine VL measurements became available in the public sector in 2015. ART regimen failure is defined as at least two VL results greater than 1000 copies /ml with good adherence to treatment in a patient who has been treated for at least six months. A VL test is mandatory to confirm diagnosis of second-line ART failure.

### Data collection and analysis: Government clinics

We conducted a cross-sectional analysis using data from all four national third-line treatment centers. We included all patients who initiated third-line therapy between 01 January 2015 and 31 December 2018. We defined third-line therapy to be any treatment regimen that included darunavir and raltegravir or dolutegravir after documented PI based second-line failure. A data collection form was used to abstract individual patient level data (Demographic, laboratory, and ART history) from clinic files at Harare and Parirenyatwa Hospitals. Data from Bulawayo were accessed using the clinic’s cohort report. The data collected included demographic variables, ART treatment history, viral load and CD4 count results.

### Data collection and analysis: Newlands clinic

We analyzed data from NC in greater detail than data from the three hospitals because of completeness. Patient data were obtained from the clinic’s electronic records. We described the population using proportions for categorical variables, medians and corresponding interquartile ranges (IQR) for continuous variables. Patients were followed from the date of third-line initiation until death, transfer, loss to follow up or data set closure, whichever occurred earlier.

We assessed the proportion of patients receiving third-line ART who achieved virological suppression (VL<1000 copies/ml) after 24 and 48 weeks. Database was closed on 31 December 2018.

### Ethical approval

This study was approved by the Medical Research Council of Zimbabwe, approval number: MRCZ/E/196. The ethics committee waived the requirement of informed consent as the study used routinely collected clinic data.

## Results

### Demographics

**Fig 1:**
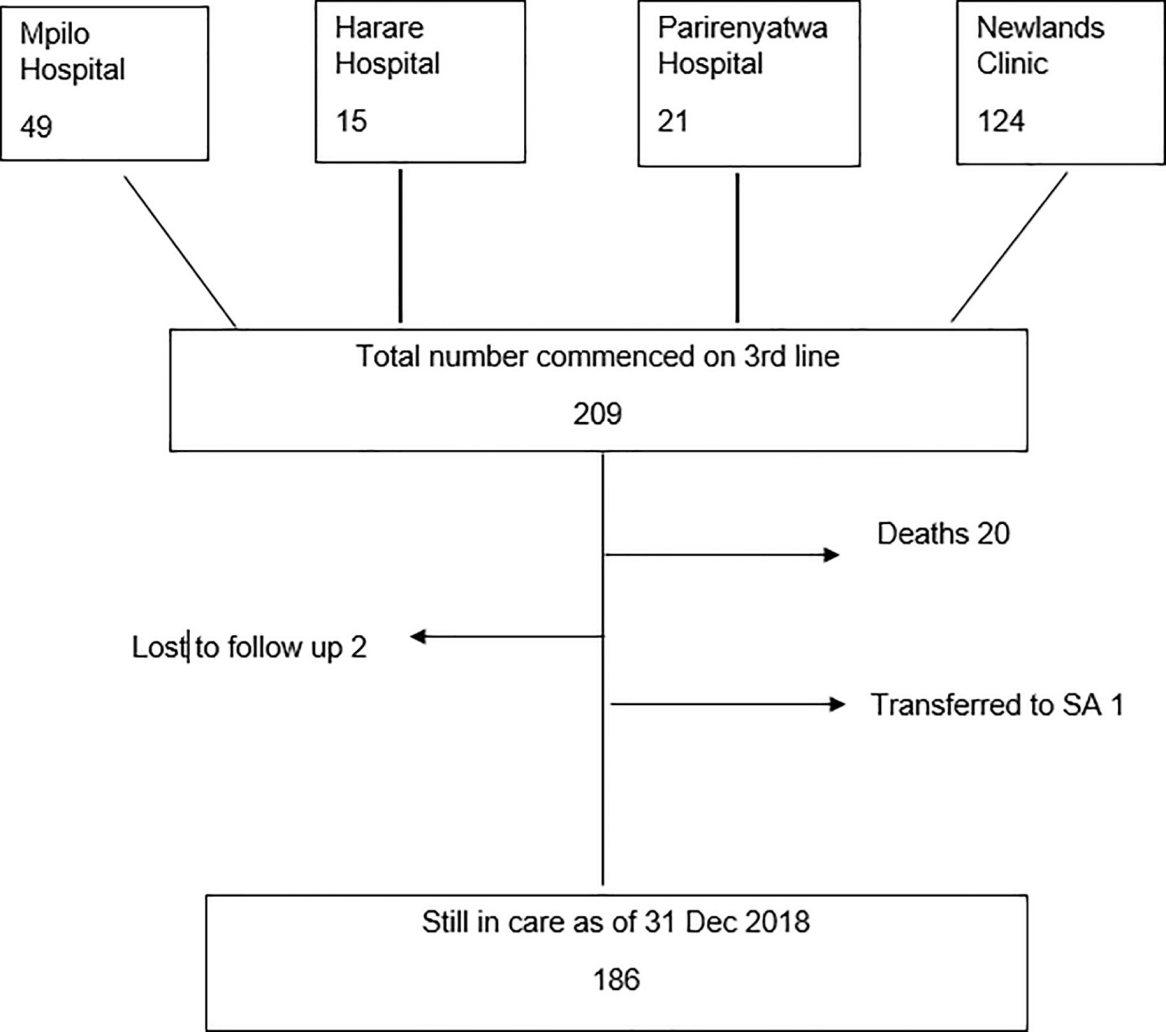
Patients commenced on third-line ART in Zimbabwe: 2015–2018.

A total of 209 patients have ever received third-line ART through the national program as of 31 December 2018. Fig 1 highlights the number of patients from the four different third-line centers. The median age at commencement of third-line ART ranged from 38 years (IQR: 20–46.5) at Harare hospital to 48.7 years (IQR:43.7–50.7) at Parirenyatwa hospital. Table 1 summarizes the sociodemographic and clinical characteristics of patients who received third-line ART at the four clinics. Only NC had complete patient level data for baseline CD4 cell count and HIV viral load. We present results in two parts. First, we summarize the results of the third-line ART programme in the 3 government (public sector) linked clinics. We then present a more detailed assessment of the third-line program at the privately-run NC.

**Table 1:** Sociodemographic and clinical characteristics of patients receiving third-line antiretroviral therapy in Zimbabwe.

Parameter: Median (IQR)	Newlands (n = 124)	Parirenyatwa (n = 21)	Harare central hospital (n = 15)	Bulawayo (Mpilo) (n = 35)**
Baseline age (yrs.)	42.1 (27.6–48.5)	48.7 (43.7–50.7)	38 (20–46.5)	44 (35.1–53.3)
Duration of first-line ART (yrs.)	4.5 (2.8–6.4)	3.3 (2.2–7)	3.5 (2.1–4.6)	5.2 (2.5–7.7)*
Duration of second-line ART (yrs.)	3.3 (2.2–6.4)	3.1 (2.8–6.2)	4.4 (2.4–5)	2.8 (1.9–4)*
Duration of third- line ART (yrs.)	1.4 (0.6–2.8)	1.3 (0.7–1.7)	1 (0.6–1.9)	2.3 (0.6–3.4)
Baseline CD4 cell count (cells/mm3)	116 (27–245)	-	-	-
Baseline viral load (copies/ml, IQR)	78 845 (17 346–256 329)	-	-	-
Number of women (%)	61 (49)	7 (33.3)	7 (46.7)	18 (51.4)

*Only 21 patients had complete data: ART = Antiretroviral therapy

** Data presented for patients with available clinic records

### Public sector patients

*Bulawayo clinic (Mpilo)*. Forty-nine patients had ever been initiated on third-line ART in Bulawayo and only 8/49 (16%) were initiated based on HIVDR test results. The HIVDR test results were not available for review. The rest were initiated after assessment by clinicians and consultations with senior doctors at the hospital. Among the 35 patients with available data on treatment duration, median duration of third line ART was 2.3 years (IQR: 0.6–3.4). Patients received second-line ART for a shorter duration (2.8 years, IQR: 1.9–4) compared to first-line ART (5.2 years). As of 31 December 2018, 7/49 (14%) had died, 3 were lost to follow up (LTFU) and 1 transferred out to South Africa.

The total number of patients in care as at 31 December 2018 was 38, and 33 of them had received third-line ART for more than 6 months. Of the 33 on treatment for more than 6 months, 25/33 (76%) had a documented VL result during the last 12 months and 22/25 (88%) had a VL below 1000 copies/ml. Two patients had low level viremia of between 200 and 1000 copies/ml.

*Harare clinics*. Thirty-six patients (22 males and 14 females) were commenced on third-line ART from the two government treatment centers in Harare (15 from Harare hospital and 21 from Parirenyatwa hospital) as of 31 December 2018. Only five patients had available documented HIVDR results and all 5 patients had triple class (PI, NNRTI and NRTI) resistant HIV. The median duration of third-line ART was 1.3 years (IQR: 0.7–1.7) at Parirenyatwa hospital and 1 year (IQR: 0.6–1.9) at Harare Hospital. Thirty-four patients had been receiving routine VL monitoring, and for 31/34 (91%) patients their most recent VL was < 1000 copies/ml after at least six months of third-line ART.

### Newlands Clinic third-line program

#### Baseline characteristics (third-line ART commencement)

Since inception of the program at NC, 124 patients (49% female) had started third-line ART. Only 9/124 (7.3%) patients commenced first-line ART at NC and the remaining 115/124 (92.7%) were transfers from other HIV treatment centers from across the nation for either first, second- or third-line ART. The median age at third-line commencement was 42.1 years (IQR: 27.6–48.5). The youngest and oldest ages at third-line commencement were 7.7 years and 71 years respectively. Median CD4 cell count and VL at third-line commencement were 116 cells/mm^3^ (IQR:27–245) and 78,845 copies/ml (IQR: 17,436–256,329) respectively. Patients had received first- and second-line ART for a median duration of 4.5 years (IQR: 2.8–6.4) and 3.3 years (IQR: 2.2–5.4) respectively.

#### HIV drug resistance

**Fig 2:**
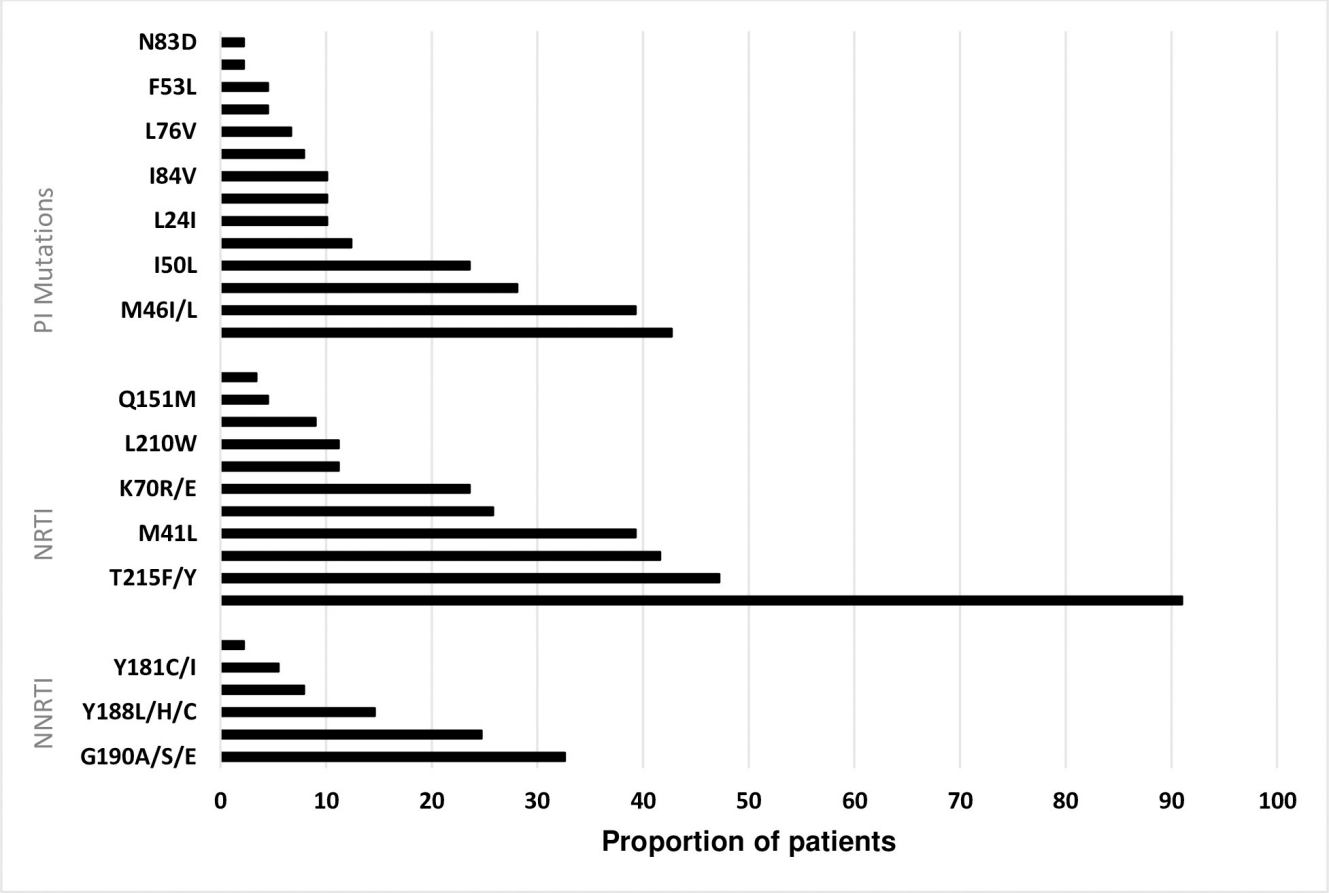
Distribution of HIV drug resistance mutations at Newlands Clinic.

A total of 89 patients (72%) had documented HIV genotyping results and 65/89 (73%) had at least one major PI resistance associated mutation (RAM). The most prevalent PI RAM was V82A/L/M/C/F which was present in 42.7% of the 89 patients. The commonest NNRTI RAM was G190A/S/E which was prevalent in 32.6% of the 89 patients and the M184V mutation was the commonest NRTI RAM present in 91% of the 89 patients. Fig 2 displays the frequency of the different RAMs. Of the patients commenced on third-line ART, 24 did not have PI resistance mutations. Unfortunately, these patients were commenced on treatment prior to receiving the HIVDR tests results due to delays. After results were received, clinicians decided to continue with the third-line regimen despite the lack of PI mutations.

#### Third-line treatment outcomes

As at 31 December 2018, 88% (109/124) of patients who commenced third line ART were still in care, 10.5% (13) had died and 1.5% (2) were transferred to other treatment centers. Median duration of third-line ART was 1.4 years (IQR: 0.6–2.8). Among the 111 patients who had received third-line ART for at least 6 months, 105 (95%) had a achieved a VL of < 1000 copies/ml [75% (83) had a VL <50 copies/ml, 16% (18) had a VL of 51–200 copies/ml, 4.5% (5) had a VL of 201–1000 copies/ml] Among the 51 patients who had week 48 VL results, 48 (94%) patients had a VL < 1000 copies/ml. One patient, a 19-year-old adolescent girl, failed third-line ART and HIVDR testing confirmed high level resistance to all protease inhibitors, reverse transcriptase inhibitors and integrase inhibitors including dolutegravir. The patient was switched to a holding regimen of zidovudine, lamivudine and abacavir and details have been published as a case report [15].

#### Mortality

At the time of death, the 13 deceased patients (7 males and 6 females) patients had received third-line ART for a median duration of 7.7 weeks (IQR: 3–23.2). The causes of death were as follows: 3 patients died of renal failure and 3 patients died of Non-Hodgkin’s Lymphoma. Other causes of death were (one patient per cause): liver failure, anemia, gastroenteritis, milliary tuberculosis, chronic pancreatitis (alcohol induced), unknown, and deep vein thrombosis. At the time of death three patients had achieved virological suppression on third-line ART.

## Discussion

We present the findings of the outcomes of the Zimbabwe national public sector third-line ART cohort. These findings are important because very few countries in sub-Saharan Africa provide third-line ART in public sector programs, hence there is scarcity of data on third-line ART in these settings. We show that there is poor access to HIVDR testing among patients failing second-line ART in the government led treatment centers. Among patients with access to HIVDR testing, there was a high prevalence of RAMs affecting PIs, NNRTIs and NRTI drug classes. Patients receiving third-line ART at NC have high virological suppression rates, with 94.5% being suppressed to below 1000 copies per milliliter. Despite the lack of complete patient level data, good treatment outcomes were achieved among patients receiving third-line in the public sector clinics.

The poor access to HIVDR testing for patients failing PI-based second-line ART in the government clinics is of great concern. While Newlands Clinic had higher rates of HIVDR testing, rates were still not 100%, which indicates a more universal issue regarding access to HIVDR testing, rather than an issue that is isolated to the public sector.

Previous studies of second-line ART outcomes in adults in SSA found that acquired resistance associated with protease inhibitors was infrequent and treatment failure was mainly due to poor adherence [7,8,10,16–18]. Clinical management of second-line failures is sub-optimal in the absence of resistance testing to identify people who harbor clinically relevant RAMs and need an optimized third-line regimen. Poor access to HIVDR testing and VL monitoring in Zimbabwe presents a very important challenge for the provision of optimum care for HIV infected patients. A simulation and cost-effective analysis projected that HIVDR testing and third-line ART would increase survival and be cost-effective in resource-limited settings compared to a public health approach of using a standard third line regimen [19,20]. Our findings of high prevalence of triple class resistance after second line ART failure in the NC cohort are consistent with previous studies [21,22]. Delays in switching patients due to poor availability of routine VL monitoring may explain the high prevalence of triple class resistance.

Our data demonstrated the effectiveness of third-line ART in a programme where the predominant subtype was C. Our findings are in line with other studies from the region that demonstrated good virological suppression rates among patients receiving third-line ART [11,12,23]. Findings from studies in resource limited settings have demonstrated that virologic suppression is a realistic endpoint for most treatment-experienced patients who begin a darunavir-based third-line therapy outside the controlled conditions of a randomized trial, at routine care settings [24–26]. NC has a very intense program to help prepare patients for third-line ART which includes a six-week enhanced adherence support program. The program focusses on identifying and addressing barriers to optimum ART adherence. Furthermore, NC also assist the very poor patients with bus fare and food to help keeping these patients engaged in care. Among the patients who died, the median duration of third-line ART was only 7.7 weeks. Early diagnosis of second-line ART failure and subsequent switch to third-line ART may help reduce mortality among these patients. The NC model of care can be replicated in the public sector clinics, however, this will require greater investment of resources in training of health workers and equipping clinics with electronic monitoring systems. We do acknowledge that that targeted retention programs (vouchers for food and transport) and electronic medical record systems at NC would have significant costs, which may not be feasible in the public sector.

The Zimbabwe national third-line ART program is facing several challenges. In our view the challenges are mainly because the program is centralized to the two largest cities in the country. This increases costs for third-line patients who are seen more frequently compared to those on first- and second-line treatment. HIVDR testing is not available in the public sector and hence patients need to access this test from the private sector at high costs unaffordable to most public health patients. Due to this prohibitive cost, management of patients is negatively affected as some end up being switched on clinical grounds without genotypic testing which overestimates those eligible and has cost implications to the program. Low VL testing coverage as reported by the national ART coordinator (44% at the end of 2018) is a challenge and impacts negatively on patient monitoring. We anticipate similar challenges in other national ART programs in SSA as countries scale up the provision of third-line ART. There is need to decentralize the provision of third-line ART to the provinces and districts to increase accessibility. Lessons can be learned from the South African third-line program were decentralization has been achieved through the aid of a well-coordinated national third-line committee [12].

The main limitations of our study are the relatively small sample size, the short duration of follow up and the missing individual patient data from the three government linked clinics. The lack of complete data regarding the number of patients failing second-line ART makes it difficult to contextualize the need for third-line ART in Zimbabwe. However, the lack of complete data is an important finding that highlights the need for the national program to invest in better and more user-friendly electronic monitoring systems. Regardless of these limitations, we find these data useful in helping national programs in the region improve care of patients post second-line ART.

## Conclusions

Our findings highlight the need for national ART programs especially those in resource limited settings to strengthen access to HIVDR testing for patients failing second-line ART. HIVDR testing is essential for the identification of patients who have acquired clinically relevant HIV resistance associated mutations, furthermore, it helps to optimize third-line regimens. This study has also demonstrated that with comprehensive care, patients who have failed on PI based second-line ART can achieve high rates of virological suppression on third-line ART. As more patients are likely to fail second-line ART, national programs must ensure that third-line ART is available through decentralization of care. Healthcare workers must be trained and equipped to identify and manage patients failing second-line therapy early.

## Supporting information

S1 Dataset (DTA)
